# Electrical burns in train climbers treated in the Helsinki Burn Centre during the last 30 years

**DOI:** 10.1186/s13049-024-01283-1

**Published:** 2024-11-12

**Authors:** Aliisa Korkiamäki, Eve Kinnunen, Andrew Lindford, Jyrki Vuola

**Affiliations:** Helsinki Burn Centre, Department of Plastic and Reconstructive Surgery, Helsinki University Hospital and University of Helsinki, P.O. Box 800, 00029 Helsinki, Finland

**Keywords:** Electrical burn, Electric train, High-voltage injury, Electric arc burn

## Abstract

**Background:**

Patients who climb onto the roof of a stationary train carriage and sustain a high voltage electrical injury from the overhead cables represent a rare type of electrical injury. The aim of this study was to review all the electrical burns and their outcomes in train climbers treated in the Helsinki Burn Centre during the last three decades.

**Methods:**

18 patients who had climbed onto the roof of a stationary electric train between November 1993 and December 2022 were included. Trauma- and outcome-related variables were collected. The primary outcome endpoints were in-hospital mortality and major amputations.

**Results:**

16 (88.9%) patients were male. The median age was 15.5 years (range: 13–29 years). All the burns were high-voltage electrical burns. The mean burn size was 45% of the total body surface area. Three (16.7%) patients died in hospital. The mean length of the Burn Centre stay was 50 days. On average, the patients underwent 5 operations (range: 0–32) during their inpatient stay. Three patients required major amputation. Eight of the patients underwent late operations. Seven (38.9%) patients exhibited late neurological dysfunction or neuropsychological symptoms at long term follow-up.

**Discussion:**

In conclusion, train climbers represent a rare group of young patients with electrical burns. Precautionary strategies should be implemented to prevent these injuries that are associated with high morbidity and mortality.

## Introduction

An electrical burn is an injury resulting from exposure to either direct current (DC) or alternating current (AC), or from a flash caused by an electric arc where electricity travels through the air. Electrical injuries are classified into two categories based on voltage: low voltage (< 1000 V) and high voltage (> 1000 V) injuries.

Direct current (DC) maintains a constant flow direction and is commonly utilized in all kinds of small handheld devices and vehicle batteries. In the context of electrical injuries, DC typically induces a single muscle contraction, which may propel the person away from the electrical source. In contrast, alternating current (AC) changes direction periodically and the frequency is measured in hertz (Hz). Low voltage AC circuits are used in household electrical systems. Direct contact with AC leads to continuous muscle stimulation, often resulting in tetanic contractions that can prevent the person from releasing the electrical source [1, 2].

In an electric arc, the electric current travels through the air without direct contact with the conductor. An electric arc can occur when a person approaches a high voltage conductor too closely. The arc can cause superficial burns due to the heat flash, or, in more severe cases, it can form a current through the person, resulting in thermal burns and internal tissue damage due to the passage of electricity. The heat flash can also cause ignition of the persons clothes and consequently cause flame burns [1, 2].

High voltage AC circuits are typically found in high-tension power lines and modern railway systems. In Finland, the electric railway utilizes AC at a voltage of 25 000 V [3]. The electricity is supplied to the trains from overhead lines via a pantograph, which collects the current from the contact wire and transmits it to the trains [4]. An electric arc originating from a railway overhead cable can reach temperatures of up to 5000 °C [5].

Even though electrical burns represent only a small number of all burns, they are associated with more severe morbidity and higher mortality compared with those of other burn aetiologies. This is evident particularly in high-voltage burns [6, 7]. Electrical burns often result in long hospital stays, repeated surgical procedures and significant financial burden to the health care [6, 8].

In Finland, there is a rare subgroup of electrical injury patients who climb onto the roof of a stationary train carriage and sustain a high voltage electrical injury from the railway overhead cables. Typically, they are young men who spend time on the rail yard during the night and then have a spontaneous idea to climb onto the roof of a stationary train. These accidents happen because of their lack of knowledge of the risks involved.

Only a few previous studies of small patient populations addressing train climbers and their outcomes have been published. This indicates that these serious injuries also occur in other countries even though the data is scarce [9–13].

In 2016, the Helsinki Burn Centre became the National burn centre for the whole of Finland, population 5.5 million. Before then, around 2/3 of severe burns in Finland were treated in the Helsinki Burn Centre. Currently, all patients with major electrical burns are referred to the Helsinki Burn Centre for further care.

The aim of this study was to review the electrical injuries and their outcomes in train climbers treated in the Helsinki Burn Centre in the last thirty years.

## Material and methods

### Study design

This was a retrospective study. Patients who had climbed onto the roof of a train and suffered a high voltage injury between April 1993 and December 2022 were included. The number of the patients who died at the scene was retrieved from the statistics of Finnish Safety and Chemicals Agency [14]. These patients never reached the hospital and therefore were not included in the study.

The primary outcome endpoints were in-hospital mortality and major amputations. Major amputation was defined as an amputation proximal to the metacarpo-phalangeal or metatarso-phalangeal joint or an amputation of multiple fingers including the thumb. Secondary outcome endpoints included the number of operations during the Burn Centre stay, minor amputations, fasciotomies, skin grafts, flap reconstructions, need for renal replacement therapy, sepsis, neurological dysfunction, length of in-hospital and intensive care unit stay, late neurological or neuropsychological symptoms as well as the need for late operations. Sepsis was defined as a generalized infection requiring treatment in the intensive care unit. Neurological dysfunction was defined as any kind of newly clinically diagnosed neurological deficit. Neuropsychological symptoms were defined as any new neuropsychological symptoms that occurred after the injury. The length of in-hospital stay was determined as the length of patients’ stay in the Helsinki Burn Centre. Further treatment in other institutions was not included in the in-hospital stay.

### Data collection

A list of consecutive patients was retrieved from the burn patient admission register of the Helsinki Burn Centre. Patients’ electronic and non-electronic medical charts were reviewed. The following variables were collected from the medical charts: demographics (age, sex, BMI, smoking, comorbidities, psychiatric disorder), trauma circumstances (date and time of the injury, the circumstances of the injury, electrical burn mechanism, voltage, flame component, suicide attempt), clinical presentation at the scene (the use of alcohol before the injury, Glasgow Coma Scale (GCS), cardiac arrhythmia, respiratory arrest), admission variables (GCS, intubation, laboratory tests such as haemoglobin, creatinine, creatine kinase and myoglobin, total body surface area (TBSA) burned, full thickness burn, clear entry/exit point, anatomical area affected by the burn, additional injuries), surgery (number and type of the operations), other outcomes (the highest level of creatinine, creatine kinase and myoglobin, renal replacement therapy, sepsis, neurological dysfunction, the length of intensive care unit and in-hospital stay, discharge to home, in-hospital death) and late outcomes (neurological dysfunction, neuropsychological symptoms, late operations).

### Statistical analysis

Because of the limited number of the patients, proper statistical analyses were not feasible. The reporting of the results is mainly descriptive. Continuous variables are reported as means, medians and ranges. Binary variables are reported as numbers and percentages.

## Results

### Patient characteristics

**Fig. 1:**
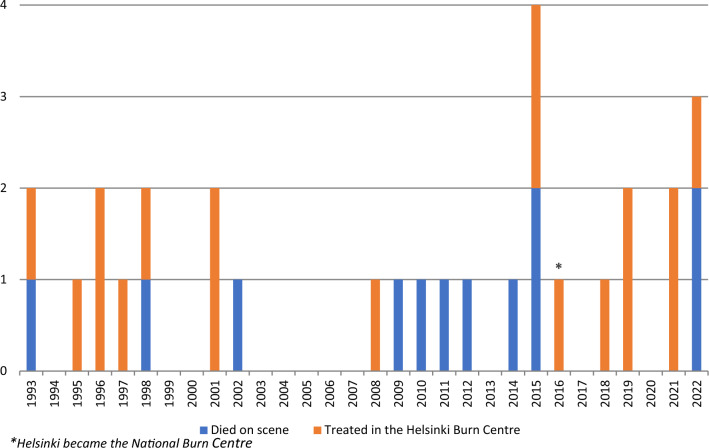
The number of train climbers suffering electrical injuries in Finland 1993–2023

During the study period there were 30 train climbers in total nationally, see Figure 1. 18 of them were treated in the Helsinki Burn Centre and were included in this study whereas the other 12 patients died at the scene. Figure 1 also shows the incidence of injuries in train climbers stratified by the year. We did not observe any significant changes over the past 30 years regarding the number or the severity of the injuries.

The patients’ characteristics are presented in Table 1. The mean age was 16.9 and median 15.5 years. The age of the patients ranged between 13 and 29 years. One of the patients was 29 years old and his was the only injury that was occupational. No patients had medications for chronic diseases apart from one patient that had medication for type 1 diabetes. Only one patient had a psychiatric diagnosis before the injury.

**Table 1:** Patient characteristics and injury information

Variable	N (%)	Mean/median (range)
Age (years)		16.9/15.5 (13–29)
Male gender	16 (88.9)	
Under the influence of alcohol	6 (33.3)	
Flame component	11 (61.1)	
Burnt area (% of TBSA)		45/40 (23–93)
Full thickness burn (% of TBSA)		19.5/17 (0–85)
Decreased GCS on scene	8 (44.4)	
Respiratory arrest	1 (5.6)	
Arrhythmia at scene	1 (5.6) *	
Intubation at scene or on admission	13 (72.2)	
Additional injuries	10 (55.6)	

*TBSA* total body surface area, *GCS* glasgow coma scale

^*^Missing data in 15/18 patients

### Injury information

The main trauma characteristics are shown in Table 1. All the injuries were high voltage electrical arc injuries and eleven of the injuries had an additional flame component. Most of the patients had burn injuries in several anatomical areas. Six of the patients had clear electrical entry and exit points. None of the injuries were a suicide attempt. Ten of the patients had additional mostly minor injuries and they are presented in Table 2. One patient had a vertebral fracture, a spinal cord injury and paraplegia.

**Table 2:** Additional injuries

Injury	N (%)
Lung contusion	6 (33.3)
Peripheral nerve injury	4 (22.2)
Pneumothorax	3 (16.7)
Vertebral fracture	2 (11.1)
Intracranial haemorrhage	2 (11.1)
Paraplegia	1 (5.6)
Clavicle fracture	1 (5.6)
Rib fracture	2 (11.1)
Kidney laceration	1 (5.6)
Facial fracture	1 (5.6)

### Outcomes

Table 3 shows the outcomes during the in-hospital stay as well as late outcomes. Fifteen patients (83.3%) required operation during the Burn Centre stay. The most common procedure was skin grafting. One patient underwent a decompression laparotomy. Eight (44.4%) patients had some sort of neurological dysfunction.

**Table 3:** Immediate and late outcomes

	Number of patients (%)	Mean (range)
*Surgery during burn centre stay*		
Number of operations		5.3 (0–32)
Skin graft	14 (77.8)	
Escharotomy	11 (61.1)	
Fasciotomy	8 (44.4)	
Local or pedicled flap	2 (11.1)	
Major amputation	3 (16.7)	
Minor amputation	0 (0)	
Microvascular flap	1 (5.6)	
*Other immediate outcomes*		
Neurological dysfunction	8 (44.4)	
Renal replacement therapy	2 (11.1)	
Sepsis	2 (11.1)	
Length of ICU stay (days)		21 (0–125)
LOS in the Burn Centre (days)		50 (1–141)
Discharge to home	8 (44.4)	
Death	3 (16.7)	
*Late outcomes*		
Neurological dysfunction	5 (27.8)	
Neuropsychological symptoms	2 (11.1)	
Late operations	8 (44.4)	
Wound-related operations	6 (33.3)	
Scar-related operations	6 (33.3)	
Microvascular flap for scar reconstruction	1 (5.6)	

*ICU* intensive care unit, *LOS* length of stay

Three patients died during the hospital stay. The deaths occurred on day 1, 8 and 125. Eight (44.4%) patients were discharged home, and the rest of survivors were discharged to another institution. Eight (44.4%) patients underwent late operations that included both wound- and scar-related operations. The injuries and main outcomes of the 18 train climbers are described in detail in Table 4.

**Table 4:** Number of required operations and major amputations during Burn Centre stay as well as in-hospital deaths in train climbers

	1	2	3	4	5	6	7	8	9	10	11	12	13	14	15	16	17	18	Mean/ N (%)
Age (years)	16	15	15	22	29	19	19	13	16	18	13	14	13	21	14	17	15	14	16.9
Burnt area (%)	37	93	50	50	39	40	43	40	41	35	40	43	92	23	32	35	55	29	45
Full thickness burn (%)	n.a	n.a	n.a	23​	35	25	20	20	n.a	1	0	14	85	8	1	0	40	1	19.5
Number of operations	2	0	4	9	4	3	2	5	3	1	0	12	32	3	4	0	9	2	5.3
Major amputation	−	−	−	+	−	−	−	−	−	−	−	−	+	−	−	−	+	−	3 (16.7)
Died	−	+	−	−	−	−	−	−	−	−	−	−	+	−	−	+	−	−	3 (16.7)

−: No

+: Yes

## Discussion

### Train surfing and train climbing phenomena

There are only a few studies that have described train-related electrical burns and possible concomitant injuries [9–11, 13, 15]. The differences of these studies are presented in Table 5. In these studies boys and young men are over-represented. In the present study also, the patient population was mainly young men with only two (11.1%) female patients. Undoubtedly, there are some innate gender related characteristics that drive young men to adventure-seeking behaviour on railway carriages.

**Table 5:** The literature review of electrical train related injuries

Author	Country	N	Year	Age	Male (%)	TBSA (%)	Amputation %	LOS (days)	Mortality rate (%)	Current	Voltage
Reichl	Britain	9	1985	13	100	39	0	40	11	AC	25 kV
Koller	Czechoslovakia	11	1991	18	91	53	0	58*	9	AC	25 kV
Sternick	Brasil	23	2000	16	100	35	9	31	44	DC	3 kV
Lumenta	Austria	12	2011	16	50	75	17**	52	8	n/a***	n/a***
Korkiamäki	Finland	18	2024	17	89	45	17**	50	17	AC	25 kV

Modified according to Table 5 in Lumenta’s study

TBSA: total body surface area burnt

LOS: length of stay

^*^Time to healing

^**^Major amputation

^***^Not mentioned in the article, but according to another reference, the electrical current used in Austrian railways system is AC at a voltage of 15 kV (https://infrastruktur.oebb.at/en/projects-for-austria/traction-current/electricity-for-railway)

The circumstances of train-related electrical injuries differ between our study and the previous ones. In our study, the patients climbed onto a stationary train carriage in the rail yard whereas in other countries such as Brazil, Austria and Germany, the patients were travelling on a roof of a moving train [11, 13, 15]. Hence, we use the term “train climbing” to distinguish the injury context from the more common “train surfing”. In previous publications, the train surfing phenomenon was related to the overcrowding of public transport but also to the sense of defiance and challenge. In our patient group, the reason for the accidents was related to spontaneous ideas or thrill-seeking and the lack of knowledge of the risks involved. None of the trains was moving during the accident.

The patients of the present study were mostly healthy with insignificant previous medical history. Only one injury was occupational whereas the rest of them (N=17, 94.4%) occurred during leisure time. Interestingly, only one third of the patients were under the influence of alcohol. These findings are comparable to Koller’s series of 11 patients [10]. In our study, the injuries typically occurred when the young patients were spending time in the rail yard and impulsively had an idea to climb onto the top of a stationary train carriage. In ten (55.6%) of the cases, a group of friends were reported to be present in the rail yard, but in all instances, only one person was injured.

### Severity of injuries

In our data, the burn injuries were severe with the mean burnt TBSA of 45% which is also in line with the previous findings varying from 35 to 75% [9–11, 13]. The highest TBSA occurred in patients who had also a flame component involved.

In previous studies [9–11, 13], the in-hospital mortality rate varied from 8 to 44% and in our study the mortality rate was 17%. The number of the patients is small in all published series and this could account for large differences in mortalities. In addition, it should be noted that during our study period there were 12 patients who died at the scene and did not reach the hospital. This would increase the mortality rate amongst train climbers in Finland even though these patients were not included in our study population.

Several studies have shown a high amputation rate after electrical burns, especially after high-voltage electrical burns [6, 16–21]. Furthermore, patients with high-voltage electrical burns are more likely to undergo amputation compared with other burn aetiologies [20]. This is mainly because of the injuries of the deep tissues and compromised limb vitality resulting from the passing electric current. In the present study, 3 patients (16.7%) required a major amputation. It is in line with Lumenta et al.’s findings with the macroamputation rate of 25% [13]. On the contrary, two studies with patient populations of 9 and 11 patients showed no amputations among train surfers [9, 10]. We could not identify any obvious reasons for the differences in the amputation rate compared with our study or Lumenta et al.’s study. Even the type of current and voltage used in electric trains was similar, at 25 kV AC, in both Reichl’s and Koller’s studies, as well as in Finland’s national railway system [3, 9, 10].

Along with the high mortality and amputation rate, the patients of the present study suffered from other significant consequences. Most of them required several operations as seen in Table 3. The intensive care unit and hospital treatment periods were lengthy and over a third of the patients even required further rehabilitation in another institution. This emphasizes the extensive and complex nature of train-related electrical injuries and was also evident in previous publications [9–11, 13].

### Late outcome

We found no previous studies describing the late outcomes of train-related electrical injuries. We were able to collect data on late outcomes which interestingly showed relatively high rates of late operations (N=8, 44.4%) including the use of a microvascular flap. In addition, our data showed that as many as seven (38.9%) of the patients had some form of late neurological dysfunction or neuropsychological symptom after the Burn Unit stay. Indeed, high rates of long-term neuropsychological symptoms have been reported after low- and high-voltage electrical injuries, the most common being sleep difficulties, anxiety and depressed mood [17]. Khor et al reported long-term neurological deficits in 16.7% of the electrical injury patients which is less than in the present study with 27.8% [20]. Furthermore, patients with electrical injuries have been found to have worse physical functioning and employment one year after the injury compared to fire or flame injuries [18]. However, in the present study we were not able to determine whether the patients were capable of returning to work as most of the patients were of pre-employment age and still in primary or secondary school.

### Effect of social media

In one recent case report, the effect of social media on the train surfing phenomenon has been discussed [22]. Because of our long study period of 30 years and social media’s rather recent arrival, we could not evaluate the effect of social media on the occurrence of train climbing-related electrical injuries. At least in one of our more recent patients, the motive for climbing on top of the train was taking a picture for social media. However, for many of the studied patients, the reasons for train climbing remained unclear and therefore the impact of social media cannot be generalized to the whole study population.

### Prevention

The main reason behind these injuries was the ignorance of the mechanisms of the high voltage arc. Considering the young age of the train climbers, the high morbidity and mortality as well as potentially life-long consequences related to their injuries, the prevention of these accidents is of utmost importance. Moreover, these injuries have a significant impact socially and economically due to the unexpected deaths and long-term disability in young people. Preventative measures include education in schools for pre-teenagers regarding the nature of high voltage current and the dangers of electric arcs and the provision of information to the parents concerning the same matters.

Preventive educational campaigns have been suggested and utilized in several countries [9–11]. In Finland, we have an ongoing social media campaign with different operators to prevent these serious injuries [23]. The campaign has been in place since 2023, so its effects cannot yet be analysed in our study.

Additionally, the development of trains over the years has led to diminished technical possibilities to climb onto the top of a train. For instance, the modern trains have smoother outer surfaces and non-opening windows which make climbing more difficult. Also, turning down the electricity in areas where trains are kept during the night would be one option. Clear warning signs, high fences and automatized surveillance systems could also help to prevent these very costly injuries.

### Limitations

The retrospective nature of this study is a major limitation. Therefore, we were not able to collect the data that was missing in some patients’ charts. Moreover, the patient population was small because of the rarity of these injuries. On the account of the limited number of the patients, we were not able to perform proper statistical analyses. Additionally, our results cannot be generalized to worldwide patient populations. Nevertheless, this study accurately describes the phenomenon in Finland.

## Conclusions

Train climbers represent a rare group of young patients with electrical burns. The injuries are associated with a significantly high morbidity and mortality. Precautionary strategies should be implemented to prevent these injuries.

## Data Availability

The anonymised data that support the findings of this study are available from the corresponding author upon reasonable request.
